# Evolutionary and plastic variation in larval growth and digestion reveal the complex underpinnings of size and age at maturation in dung beetles

**DOI:** 10.1002/ece3.8192

**Published:** 2021-10-14

**Authors:** Patrick T. Rohner, Armin P. Moczek

**Affiliations:** ^1^ Department of Biology Indiana University Bloomington Indiana USA

**Keywords:** gut retention time, life history, nutritional plasticity, *Onthophagus*

## Abstract

Age and size at maturity are key life‐history components, yet the proximate underpinnings that mediate intra‐ and interspecific variation in life history remain poorly understood. We studied the proximate underpinnings of species differences and nutritionally plastic variation in adult size and development time in four species of dung beetles. Specifically, we investigated how variation in insect growth mediates adult size variation, tested whether fast juvenile growth trades‐off with developmental stability in adult morphology and quantified plastic responses of digestive systems to variation in food quality. Contrary to the common size–development time trade‐off, the largest species exhibited by far the shortest development time. Correspondingly, species diverged strongly in the shape of growth trajectories. Nutritionally plastic adjustments to growth were qualitatively similar between species but differed in magnitude. Although we expected rapid growth to induce developmental costs, neither instantaneous growth rates nor the duration of larval growth were related to developmental stability in the adult. This renders the putative costs of rapid growth enigmatic. We further found that larvae that encounter a challenging diet develop a larger midgut and digest more slowly than animals reared on a more nutritious diet. These data are consistent with the hypothesis that larvae invest into a more effective digestive system when exposed to low‐quality nutrition, but suggest that species may diverge readily in their reliance on these mechanisms. More generally, our data highlight the complex, and often hidden, relationships between immature growth and age and size at maturation even in ecologically similar species.

## Graphical abstract

Age and size at maturity are key life‐history components, yet the proximate underpinnings that mediate intra‐ and interspecific variation in life history remain poorly understood. Combining detailed assessments of nutritional plasticity in larval growth and digestion, our data highlight the complex, and often hidden, relationships between immature growth and age and size at maturation even in ecologically similar species.

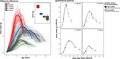

## 1 INTRODUCTION

Body size is tightly related to an individual's fitness and thus represents a key life‐history component (Roff, 2002; Stearns, 1992). Consequently, the ultimate selective forces shaping evolutionary and plastic responses of body size have been studied extensively and are, by now, relatively well characterized (Blanckenhorn, 2000; Honek, 1993; Peters, 1986). However, the diverse mechanisms that mediate variation in growth across levels of biological organization remain poorly understood outside select model systems (e.g., *Drosophila melanogaster* [Texada et al., 2020]). This limits our understanding of how variation in adult size arises, whether the same mechanisms act across species and environments, and to what extent differences in juvenile growth and physiology limit or facilitate the evolution of adult size. Collectively, this hampers our understanding of the mechanistic underpinnings of life‐history evolution (Flatt & Heyland, 2012).

A major source of variation in adult size is mediated by plasticity in response to variation in nutritional quality and quantity (Blanckenhorn, 1999; Stillwell et al., 2010; Teder & Tammaru, 2005). Ontogenetic responses to nutrient limitation may emerge as simple byproducts of the physiological demands of development. Alternatively, they may also reflect evolved responses for utilizing nutritional cues as a source of regulatory information to then mediate adaptive adjustments of juvenile development to environmental conditions (Sultan, 2015). For example, if an ephemeral larval food source is depleted, the premature abbreviation of larval growth can allow individuals to escape ephemeral larval habitats before these are fully depleted (these are common responses in amphibians: Newman, 1992; flies: Blanckenhorn, 1998; and beetles: Shafiei et al., 2001).

Both evolutionary and plastic responses of body size to nutritional variation have been studied extensively in insects (Whitman & Ananthakrishnan, 2009). This work has revealed that insect body size is not a simple function of growth rate over time. Instead, insect growth emerged as discontinuous and heavily context‐dependent (e.g., Parker & Johnston, 2006). By focusing on composite measurements such as egg‐to‐adult development time and growth rates, the mechanistic link between growth and adult size remains indirect and obscure (Dmitriew, 2011; Rohner et al., 2017; but see: Davidowitz, 2016). This has several implications. Firstly, it remains unclear precisely which aspects of larval growth dynamics underpin canalized and plastic size responses to environmental variation. This complicates the assessment of whether similar mechanisms act in different species and prevents linking findings from evolutionary and ecological studies to our growing understanding of the developmental and physiological regulation of insect growth (Nijhout et al., 2014). Secondly, as the source of variation in adult size often remains unknown, this complicates assessing trade‐offs between growth and adult performance. For instance, it has been hypothesized that rapid growth can lead to negative effects later in life, such as decreased developmental stability (Dmitriew, 2011; Schäfer et al., 2013). Yet, testing these hypotheses requires detailed assessments of variation in growth. Thirdly, the behavioral and physiological mechanisms that mediate interspecific differences and plastic variation in larval growth remain poorly understood. For example, changes in resource acquisition by adjustments of foraging behavior, gut morphology, or digestive efficiency may individually and interactively contribute to canalized or plastic variation in growth (Sassi et al., 2007; Wagner et al., 2009); yet beyond select model systems, the mechanistic means by which immature insects develop and evolve to confront a nutritionally variable environment remain unclear, hampering our mechanistic understanding of life‐history evolution and trade‐offs.

Here, we investigate juvenile growth dynamics and their relationship to age and size at maturation in four species of dung beetles (Scarabaeinae) of varying body sizes: the relatively large *Digitonthophagus gazella*, the intermediate‐sized *Onthophagus binodis*, and the much smaller *Liatongus militaris* and *Onthophagus taurus*. These species are particularly interesting to study in this context because they strongly differ in adult size yet have similar feeding ecologies and reproductive behaviors, limiting the confounding effects of trophic level and physiological constraints on size and growth (e.g., Clauss et al., 2003; Cohen et al., 1993; Gittleman, 1985). Specifically, all four species are paracoprid nesters, meaning that they all dig tunnels directly underneath cow pats that accommodate several brood chambers filled with cow dung (so‐called “brood balls”). In each of these brood balls, females deposit a single egg and do not provide any further maternal care (as is the case in other dung beetles; Hanski & Cambefort, 1991). This reproductive behavior has several implications for the relationship between larval growth and adult size. Firstly, cow dung is a challenging diet rich in hard‐to‐digest polysaccharides such as cellulose and low in nutrients such as essential amino acids (Holter, 2016). Secondly, a larva's brood ball represents its only source of nutrition throughout its entire juvenile development. The size and quality of the brood ball therefore directly affect growth outputs (Moczek, 1998) and because mothers often compete for food when constructing brood balls, larvae are often subjected to nutrient limitation. Thirdly, being physically confined to their brood ball, dung beetle larvae lack the ability to forage selectively on different food sources to optimize nutrient uptake. This is in sharp contrast to other, more mobile insects such as caterpillars (Waldbauer et al., 1984) or juvenile cockroaches (Raubenheimer & Jones, 2006). Dung beetle larvae may thus be expected to evolve particularly strong plastic responses to nutritional conditions.

In addition to their similar feeding ecology, all four beetle species investigated here are common members of agricultural grassland communities across the globe. While *D*. *gazella*, *O*. *binodis*, and *L*. *militaris* are native to sub‐Saharan Africa, *O*. *taurus* is native to the Mediterranean region and Central Europe. However, all four species have been introduced purposefully and/or accidentally to other locations, including south‐eastern Australia where all four species currently co‐occur (see Figure S1 for distribution maps).

In an effort to uncover the proximate mechanisms underpinning variation in the age and size at maturation, we use experimental manipulation of nutritional quality in these four species to assess in detail various aspects of larval growth. We first determined canalized and plastic developmental mechanisms contributing to variation in adult body size within and among species. Next, we investigated putative costs of the large variation in larval growth in later life stages by testing whether rapid immature growth trades off with developmental stability in the adult. Finally, we investigated the mechanistic basis of evolved and plastic responses of digestive systems in dung beetles by applying a newly developed assay to assess plasticity in gut morphology as well as gut residence time in the two species with the most divergent growth schedules. We discuss the complex, but often hidden, relationship between juvenile growth and age and size at maturation, demonstrate that plasticity in larval growth can underpin robustness in adult size, and highlight the role of plasticity in digestive systems in growth and life history.

## 2 MATERIALS AND METHODS

To study plasticity and evolutionary dynamics of larval growth, we simultaneously reared four ecologically similar species of dung beetles under strictly controlled laboratory conditions. All four species are tunneling scarabs, found commonly on pastures, have been the subject of repeated accidental and deliberate introductions, and co‐occur in either their native or introduced ranges, or both (reviewed in Pokhrel et al., 2021). For the purposes of this study, *Digitonthophagus gazella* (Fabricius, 1787) was collected in Santa Fe (Florida, USA); *Onthophagus binodis* (Thunberg, 1818) stem from Waimea (Hawaii, USA); *Onthophagus taurus* (Schreber, 1759) was collected in Santa Fe (Florida, USA) and Michigan (Michigan, USA); and *Liatongus militaris* (Castelnau, 1840) originated from Imbil (Queensland, Australia). Wild‐caught individuals were shipped to Bloomington, Indiana, where laboratory colonies were established following standard procedures (Shafiei et al., 2001).

### 2.1 Evolutionary and nutritionally plastic responses of larval growth

To assess larval growth trajectories, we haphazardly selected 4 to 6 laboratory‐reared females from each of our laboratory colonies and transferred them into rectangular oviposition containers (27 cm × 17 cm × 28 cm) that were filled with a sterilized sand–soil mixture and topped off with ca. 800g defrosted cow dung (this rearing method has been applied previously in these species: Beckers et al., 2015; Macagno et al., 2018; Rohner, 2021). Because morphological and life‐history traits are strongly dependent on larval nutrition and maternal investment (Moczek, 1998), we removed eggs from their natal brood balls and reared them in standardized, artificial brood balls as described previously (Shafiei et al., 2001). In brief, we opened all natal brood balls and transferred eggs or newly hatched first instar (L1) larvae (before they started feeding) into separate wells of a standard 12‐well tissue culture plate. Each well was filled with 3.2 g feeding substrate, which corresponds to an ad libitum food treatment for even the largest beetles in our study. As feeding substrate, each well was provisioned with one of two types of cow dung. Half of all individuals were reared on high‐quality dung produced by cows that had been fed grass exclusively, while the other half was provisioned with low‐quality dung derived from hay‐fed cows. Hay‐ versus grass‐derived cow dung differs in nutritional quality, texture, and consistency, yet both dung types are readily utilized by all four species in the field. A large quantity of each dung type was collected at the beginning of the study, thoroughly mixed using a hand‐held electric cement mixer (Nordstrand, PWT‐PM0), frozen in separate aliquots, and then thawed for larval rearing as needed.

Larvae in 12‐well plates were kept at constant 27°C. Initially, larvae were checked every 24 h to record their developmental stage (egg; larval instars L1, L2, and L3; pupa; adult) and survival. We recorded the larval age at the first day of each larval instar and weighed larvae using a Mettler Toledo (AL54 Ohio, USA, *d* = 0.1 mg) scale. Once larvae reached their third instar, individuals were weighed every 48 h until pupation. Pupal weight was assessed four days after pupation. Adult eclosion was recorded and animals were collected, sacrificed, and stored in 70% ethanol 4 days after emergence. Pronotum width was used as estimate of adult body size. Based on these data, we calculated egg‐to‐adult development time, larval peak weight, and the age at which larvae reached their peak weight. As an estimate of instantaneous growth rate for each individual, we used linear regressions to estimate the increase in logarithmized larval mass per day during the first four days of the third instar. This is a time span in which larvae of all species grow at their maximal rate. In addition, and for comparison, we also computed the commonly used “integral” growth rates (see: Tammaru & Esperk, 2007), that is, simple ratios between adult body size over total egg‐to‐adult development time.

We first tested for differences in survival across species and treatments using a generalized linear model with binomial error distribution, using the function *glm()* as implemented in R v3.6.3 (RCoreTeam, 2020) with type II sums of squares as implemented in the R package *car* (Fox & Weisberg, 2018). Next, because larval growth was complex and did not fit any commonly used growth function (e.g., Gompertz, von Bertalanffy, or logistic models), we used generalized additive models (GAMs; as implemented in the R package *mgcv* [Wood, 2011]) to test for differences in the shape of growth trajectories. This very flexible type of linear model uses a combination of smoothing functions to fit complex nonlinear relationship between dependent and independent variables. In our case, we used thin plate regression splines to model larval weight as a function of age. We then used the Akaike information criterion (AIC) to assess whether a GAM including treatment and species effects fit the data better than a similar model only including either of the terms alone. In addition, for each species, we tested for a treatment effect while fitting separate growth curves for each individual as random effect.

Next, we assessed whether the relationship between major aspects of larval growth and adult size varies between species and nutritional context. To this end, we used linear models (type II SS) to fit body size as a function of species, treatment, and one of several variables of interest (egg‐to‐adult development time, the duration of larval growth, log peak weight, log weight loss, log pupal weight, growth rate). Specifically, we tested for interactions between aspects of growth and species or treatment, which would indicate that that the relationship between larval growth and adult body size depends on the evolutionary or environmental context.

### 2.2 Trade‐off between rapid growth and developmental stability

Comparing growth trajectories revealed large variation in growth rates as well as in the duration a larva remains in the third instar (L3) following the cessation of growth. We sought to determine whether the speed or duration of growth correlates with measures of developmental stability in the resulting adults. To test whether rapid juvenile development causes elevated developmental instability in the adult, we quantified fluctuating asymmetry (FA; a form of developmental instability [Klingenberg & McIntyre, 1998]) in the adult fore tibia. This trait was chosen because tibial primordia undergo rapid cell proliferation and differentiation during L3, and as the primary digging appendage of tunneling scarabs, it represents a functional trait putatively under selection in the adult (see Linz et al., 2019; Macagno et al., 2016; Rohner & Moczek, 2020).

To quantify adult tibia shape, we followed previously established protocols (Rohner et al., 2020). In brief, we removed both fore tibiae and photographed them using a digital camera (Scion, Frederick, MD, USA) mounted on a Leica MZ‐16 stereomicroscope (Bannockburn, IL, USA). Two‐dimensional landmarks for 9 full landmarks were acquired using tpsDig2 (Rohlf, 2009). Landmark coordinates were subjected to a full Procrustes superimposition using the function *gpagen*() of the R package *geomorph* version 3.1.1 (Adams et al., 2020). As an estimate for fluctuating asymmetry (FA) in shape, we calculated the Procrustes distance (i.e., the square root of the sum of squared differences in landmark positions) between the left and right tibia of each individual. To quantify FA in size, we divided the absolute difference in centroid size between the left and right tibia by the average of the left and right side. To test for trade‐offs between growth and FA, we fitted FA in size or shape as a function of growth rate and the duration a larva spent in L3 after reaching its peak weight. Measures of FA for centroid size and shape were square root transformed to better approximate normality of residuals.

### 2.3 Plasticity of digestive systems

**FIGURE 1:**
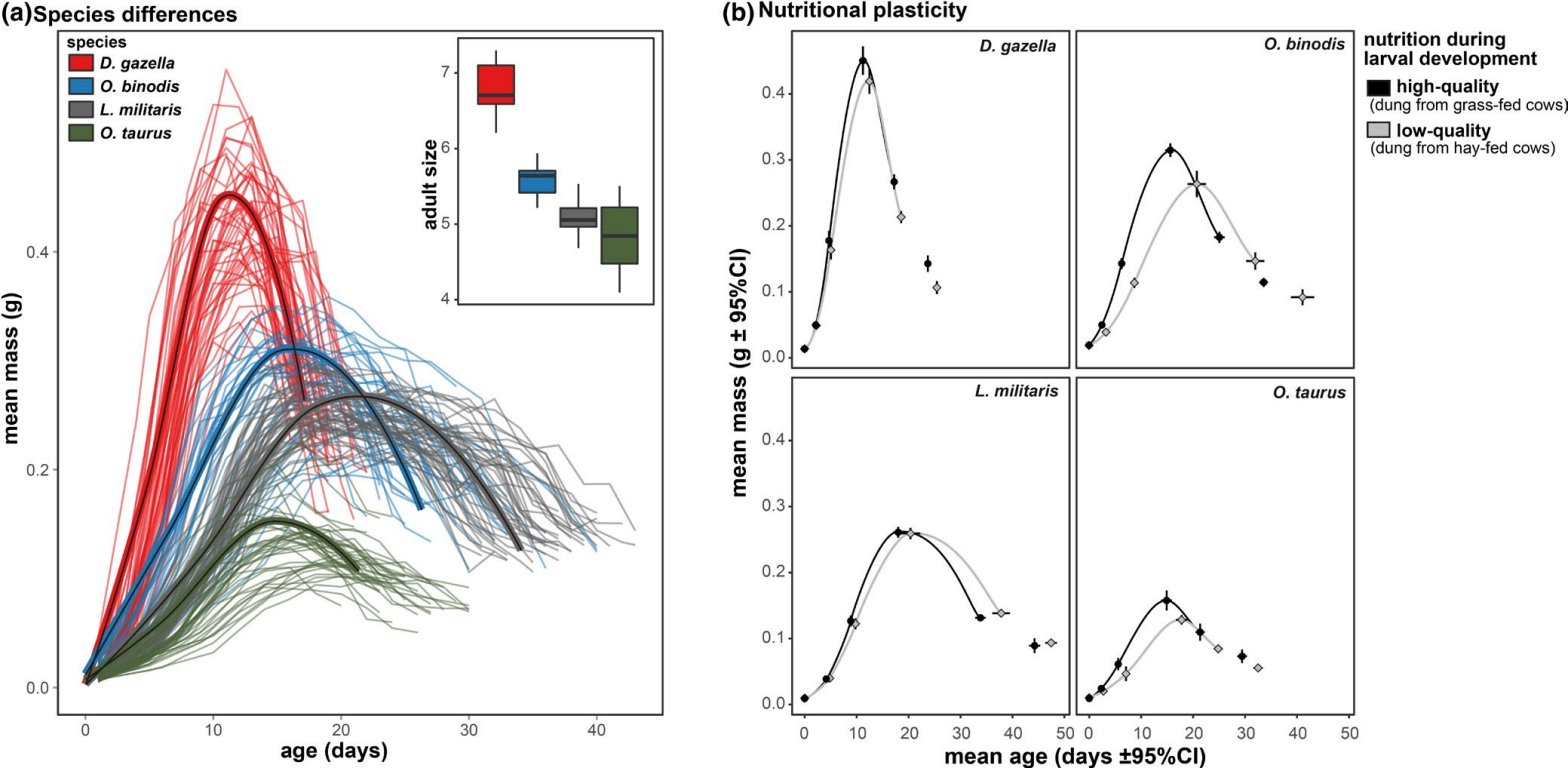
Nutritionally plastic and evolved differences in juvenile growth curves. Panel (a) illustrates individual and species differences. Lines represent individual larval weight trajectories, while pupal and adult weights are not shown. Bold lines represent loess smoothing functions based on stage‐specific means for each species separately. Panel (b) shows species‐specific nutritional plasticity in larval growth trajectories when fed on high‐ and low‐quality diets. Means (±95% CIs) refer to average age and weight at six specific developmental stages: the onset of the larval stages L1, L2, L3, the time point when larvae reach peak weight, pupation, and adult emergence. Loess smooth functions are based on stage‐specific means

To investigate nutritional plasticity in gut morphology and digestive physiology in dung beetle larvae, we first reared the two species with the strongest differences in adult size, the small *O*. *taurus* and the large, fast developing *D*. *gazella* (Figure 1), on high‐ and low‐quality cow dung (as above). Once larvae reached their peak weight, larvae were weighed (using a Mettler Toledo scale; AL54 Ohio, USA, *d* = 0.1 mg), sacrificed in 96% ethanol, and dissected in 0.05% Triton‐X in phosphate‐buffered saline solution. Dissected intestinal tracts were photographed using a digital microscope (Crenova). In the analysis, we chose to focus on midgut size because in contrast to the fore‐ and hindgut, the midgut is not lined with cuticle and plays a major role in the secretion of digestive fluids and the absorption of nutrients from the lumen (Snodgrass 1994). When dissected, the midgut also represents a simple cylindrical tube that can be described with simple length and width measurements (using the “segmented line” tool in ImageJ [Schneider et al., 2012]). Midgut volume was calculated using the formula of the volume of a cylinder (volume = (midgut width × 0.5)^2^ × *π* × midgut length). We used linear models (type II SS) to test for species and treatment differences on log midgut length, width, and volume using log larval mass as a covariate.

In addition to nutritional plasticity and species differences in midgut size, we were also interested in whether larvae adjust gut retention time (i.e., the time needed for a food item to pass through the intestinal tract). To this end, we again reared larvae of *D*. *gazella* and *O*. *taurus* on high‐ and low‐quality cow dung as described above. Once larvae reached their third instar, we performed feeding trials. During the trial, half of all larvae were provided with low‐quality nutrition while the other half was fed high‐quality dung. That is, we crossed a nutritional treatment administered during larval development (nutrition during development) with another nutritional treatment used during the trial (nutrition during trial). To track food administered during the trial period, we mixed fine‐grained vermiculite into the cow dung. Vermiculite is a soft and inert mineral often used in horticulture and has previously been used to distinguish between different dung types consumed by beetles and in analyses of maternal brood ball parasitism (Moczek & Cochrane, 2006). Adding this material into the larval food allowed us to tell apart gut content consumed just before the trial period (no vermiculite) from food consumed just after (now containing vermiculite). After 1.5 h, all larvae were sacrificed, dissected, and photographed as described above. The position of the vermiculite particles that passed the farthest through the intestinal tract relative to the length of the fore and midgut was recorded. Dividing the duration of the feeding trial (1.5 h) by the ratio between the distance vermiculite has traveled in the gut and total gut length yields the expected gut retention time. Because these feeding trials were repeated in different temporal replicates (blocks), we used linear mixed models with block as random effect in our analyses to test for species differences, the effect of nutrition during development, and nutritional treatment administered during the trial on gut retention time.

## 3 RESULTS

Using detailed observations of larval growth throughout ontogeny, we sought to quantify evolutionary and plastic responses of insect growth and its relationship to adult size. Of the 255 larvae reared in artificial brood balls, 175 individuals (68.6%) successfully reached their adult stage. Larval survival was higher for *L*. *militaris* (0.83 [0.72, 0.91] 95% CI) than for the other species (*D*. *gazella*: 0.68 [0.56, 0.77], *O*. *binodis*: 0.64 [0.51, 0.76], *O*. *taurus*: 0.60 [0.48, 0.71]), but there was no significant difference in survival between animals reared on grass versus hay dung (nutritional treatment × species‐interaction: *Χ* ^2^_(3)_ = 5.09, *p* = .165; treatment effect: *Χ* ^2^_(1)_ = 0.03, *p* = .861).

### 3.1 Evolutionary and nutritionally plastic responses of larval growth

**FIGURE 2:**
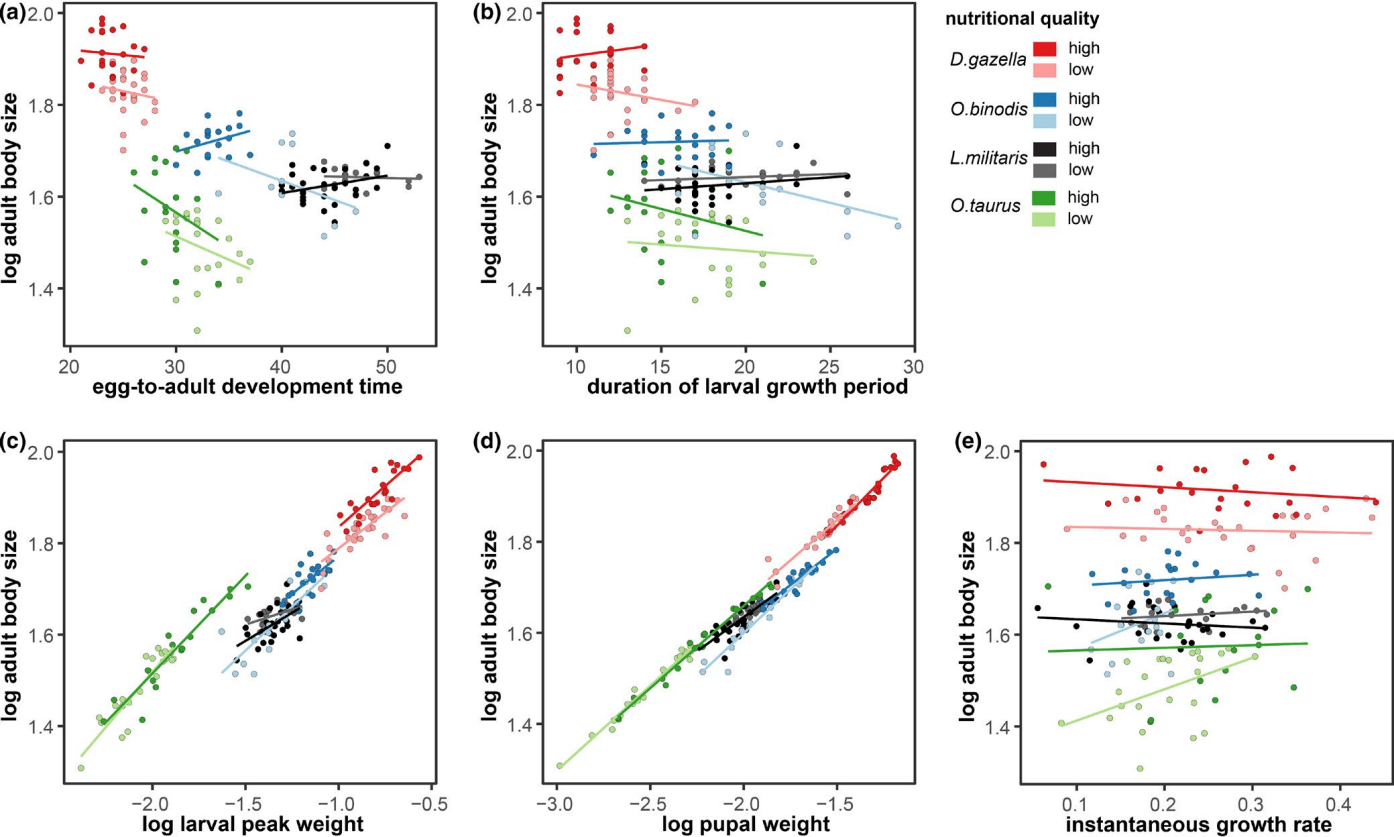
Species differences and nutritional plasticity in the relationship between different aspects of larval growth, development, and body size

The relationship between juvenile development time and adult size differed strongly and, unexpectedly, between species (Figure 2a). Although *D*. *gazella* is the largest of the four species investigated here (adult pronotum width: 6.48 mm (± 0.06 SE)), it had by far the shortest development time (24.7 days ± 0.23 SE). This is in sharp contrast to *L*. *militaris*, which is much smaller (5.12 mm ± 0.02 SE) yet took on average an additional 21 days (average: 45.6 ± 0.45 SE) to complete immature development. Developmental times of the other two species were roughly intermediate, despite major differences in average body sizes: *O*. *binodis* required 36.3 days (± 0.06 SE) on average to reach an adult size of 5.36 mm (± 0.06 SE), whereas the smallest of the four focal species, *O*. *taurus* (4.60 mm ± 0.07 SE) required 31.2 days (± 0.41 SE) to mature into an adult. A similar negative relationship between adult body size and egg‐to‐adult development time was also evident across nutritional treatments within species in *D*. *gazella* (*F*_1,46_ = 20.45, *p* < .001), *O*. *binodis* (*F*_1,26_ = 7.08, *p* = .013), and *O*. *taurus* (*F*_1,37_ = 18.75, *p* < .001). In contrast, this relationship was weakly positive in *L*. *militaris* (*F*_1,46_ = 4.06, *p* = .050). The four species investigated therefore do not follow the otherwise widespread and positive intra‐ and interspecific relationship between development time and adult size (Roff, 2002).

Species differences in the overall shape of larval growth trajectories were also readily apparent (Figure 1). *Digionthophagus gazella* exhibited the fastest mass gain during early development and reached its peak weight very early compared to the other species, but lost about half of its mass between larval peak weight and pupation. In contrast, *O*. *binodis* and *L*. *militaris* exhibited much slower growth rates early in development and reached peak weight later, with peak weights plateauing for much longer compared to the average trajectories of *D*. *gazella*. Lastly, *O*. *taurus* emerged as the species with the slowest growth rate and the smallest adult body size (Figures 1 and 2e).

**FIGURE 3:**
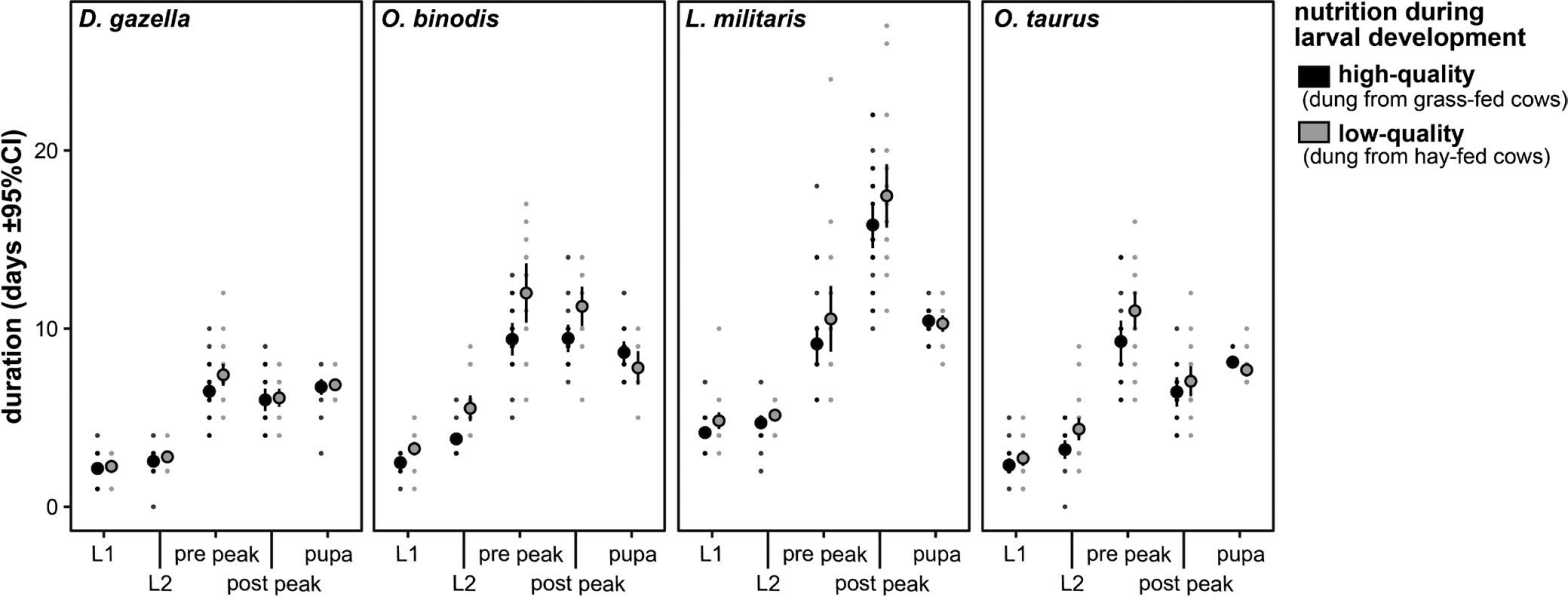
Average time spent in each developmental stage when reared under high‐ and low‐quality nutritional conditions. The duration of the third larval instar has been separated into the period before reaching peak weight (prepeak) and after peak weight (postpeak). Note that species differ greatly in the time it takes third instar larvae to reach peak weight *relative* to the time it takes those same larvae to then reach pupation. Error bars represent 95% confidence limits (*n* = 175)

Nutritional stress reduced adult body size in all species except the slow‐growing *L*. *militaris* (species‐by‐treatment interaction: *F*_3,165_ = 10.55, *p* < .001). Yet, the duration of the immature stages and growth rates were affected in all species (Figures 1 and 3; Table S1). However, these effects were much stronger in *O*. *binodis* than in any of the other species (e.g., duration of L3: species‐by‐treatment interaction: *F*_3,170_ = 3.57, *p* = .015). That is, even though nutritional plasticity was found in all species and caused qualitatively similar changes to their larval growth schedules, it differed strongly in its magnitude. Evolved differences in nutritional plasticity were further tested using generalized additive models. Including a species‐by‐treatment interaction greatly increased the model fit compared to a model only including main effects of species and treatment (ΔAIC = 320.6), suggesting species‐specific nutritional responses. When taking individual variation into account (by adding each individual's growth curve as random effect), treatment effects were also significant when analyzing each species separately (all ΔAIC > 2).

**FIGURE 4:**
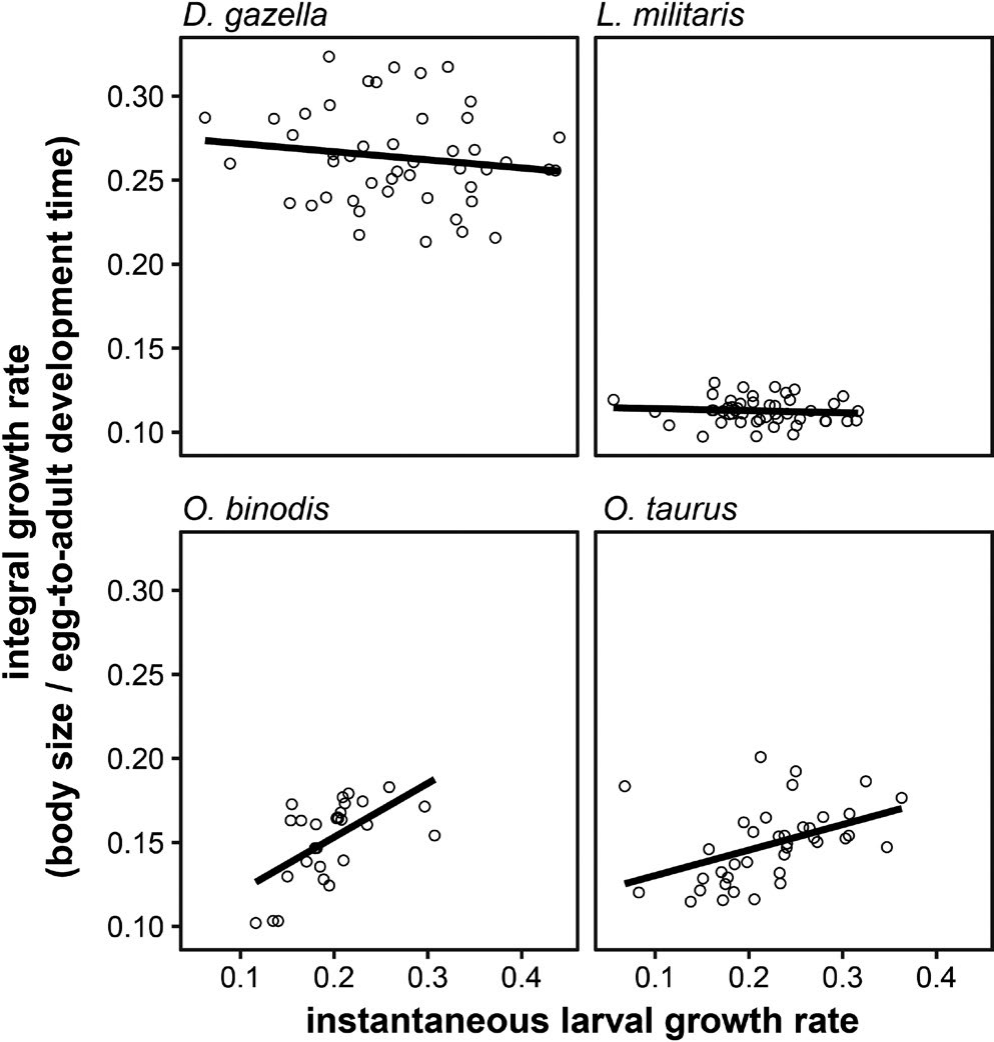
Integral growth rate (simple adult size/egg‐to‐adult development time ratio) plotted against instantaneous growth rate during the early third instar. While *O*. *binodis* and *O*. *taurus* show a positive relationship as expected, *D*. *gazella* and *L*. *militaris* do not show any significant relationship

Lastly, our data also allowed us to test for species differences in the relationship between aspects of growth and adult size (Figure 2). Larval peak weight was a strong predictor of adult size, but this relationship differed between species: *D*. *gazella*, *O*. *binodis*, and *L*. *militaris* larvae needed to reach heavier peak weights to reach the same adult size as the much smaller *O*. *taurus*. Similar patterns were found for pupal weight. Although heavier pupae gave rise to larger adults, this relationship was again species‐specific. Likewise, the relationship between growth rate and egg‐to‐adult development time also tended to have a species‐specific relationship with adult body size (*p* < .1; Table S2). In addition, instantaneous growth rates correlated with (integral) mass‐over‐time ratios only in the two *Onthophagus* species but not in *D*. *gazella* and *L*. *militaris*, underscoring that simple ratios derived from adult size and total development time may be poor predictors of larval growth dynamics (Figure 4). Taken together, these findings suggest that variation in adult body size is caused by complex species‐ and environment‐dependent adjustments of larval growth trajectories.

### 3.2 No evidence for a trade‐off between rapid growth and developmental stability

Considering the large variation in larval growth schedules, we tested whether rapid larval development trades off with developmental stability in the adult. We found that fluctuating asymmetry in size and shape of the adult fore tibia were independent of species identity and nutritional treatment, and neither correlated with growth rate, nor the time spent in L3 after the cessation of growth (all *p* > .05; Table S3; Figure S2). Our results therefore fail to support the hypothesis that rapid growth during the juvenile stages trades off with developmental stability in simultaneously developing structures.

### 3.3 Gut length and retention time are nutritionally plastic

**FIGURE 5:**
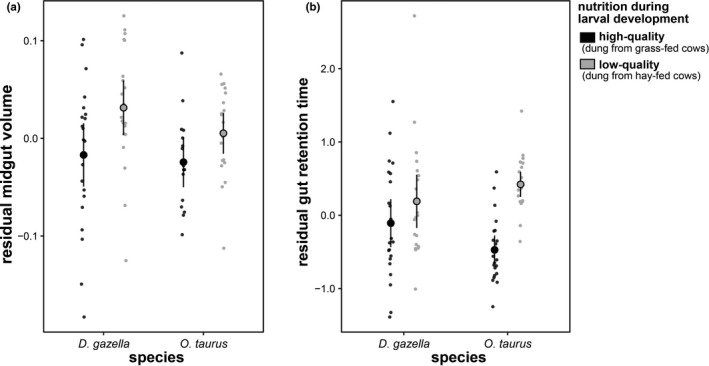
Evolutionary divergence of, and nutritional plasticity in, digestive systems. (a) *D*. *gazella* as well as *O*. *taurus* develop larger midguts in response to low‐quality nutrition (residuals are derived from a linear model of log midgut volume on log pronotum width). (b) Beetle larvae that were exposed to low‐quality nutrition took longer to digest a meal. To control for differences in substrate consistency, feeding trials were performed with high‐ and low‐quality food. Because the nutritional quality of the food (dung) used during the feeding trials did not affect gut retention time, both groups (low‐ and high‐quality) were combined in panel (b). Error bars indicate 95% confidence intervals

Lastly, we sought to shed light on the physiological and developmental processes that may enable larvae to adjust growth dynamics in a nutrition‐ and species‐specific manner, by assessing the potential role of plastic responses and evolutionary differentiation in gut size and gut retention time. Larvae of the small and relatively slower growing *O*. *taurus* and the large and rapidly growing *D*. *gazella* both developed disproportionately large midguts when reared on a nutritionally challenging diet (treatment effect on log midgut volume: *F*_1,75_ = 4.62, *p* = .035; Figure 5a). While this effect was mediated by an increase in midgut width in *O*. *taurus* and *D*. *gazella* (treatment effect: *F*_1,73_ = 4.34, *p* = .041), only the latter also showed nutritional plasticity in midgut length (species‐by‐treatment interaction: *F*_1,73_ = 6.93, *p* = .010). This indicates that digestive morphology is nutritionally plastic and that species may diverge in the degree and nature of this plasticity.

Likewise, the exposure to low‐quality nutrition during larval development increased gut retention time in both species, although this effect was stronger in *O*. *taurus* (nutrition during development‐by‐species interaction: Χ^2^_(1)_ = 13.43, *p* < .001; Figure 5b). In contrast, whether high‐ or low‐quality dung was fed to larvae during the actual experimental trial did not affect retention time (Χ^2^_(1)_ < .01, *p* = .964; not shown). This suggests that retention time is not a function of short‐term effects related to food structure or consistency, but that nutrition experienced throughout early development causes more long‐term developmental responses in the digestive physiology of larvae. Taken together, these findings indicate considerable developmental plasticity in gut retention time and gut size consistent with the hypothesis that larvae invest into a more effective digestive system when exposed to low‐quality nutrition, suggesting that species may diverge readily in their reliance on these mechanisms.

## 4 DISCUSSION

Our comparison of nutritional plasticity of larval growth and gut physiology across dung beetle species rendered four salient results. Firstly, larval growth trajectories differ widely between species in a manner that is inconsistent with the trade‐off between development time and adult size otherwise common in insects occurring in ephemeral habitats. Second, nutritional plasticity in growth dynamics was ubiquitous and diverged among species mainly in its magnitude. The relationship between larval growth and adult size is therefore strongly species‐ and environment‐specific. Thirdly, variation in growth rates and the duration of growth are not correlated with fluctuating asymmetry in the shape or size of adult fore tibiae. We thus find no evidence for a trade‐off between larval growth and developmental stability. Finally, nutritional body size plasticity coincided with plastic responses in midgut size and gut retention time. These findings are consistent with the hypothesis that nutritional plasticity in digestive systems contributes to adaptive adjustments to growth schedules. Below, we discuss how developmental plasticity in larval growth and digestive systems shape the evolutionary ecology of dung beetle life history.

### 4.1 Context‐dependent relationship between growth, size, and age at maturation

Evolutionary changes in the age and size at maturation necessitate adjustments of the developmental mechanisms underpinning growth. Yet, how growing systems modify their own maturation to match developmental outputs to external conditions remains poorly understood outside select model systems (e.g., the tobacco hornworm *Manduca sexta* [Grunert et al., 2015; Nijhout et al., 2014]). We show that four closely related species with similar feeding ecology differ substantially in how key aspects of larval growth contribute to adult size (Figures 1 and 2). For instance, larger bodied species are generally expected to take longer to reach the adult stage (Ricklefs & Wikelski, 2002; Stearns, 1992). However, the largest of the four species studied here, *D*. *gazella*, reached the adult stage fastest, enabled by relatively fast growth rates during a comparatively short period of growth that culminated in high larval and pupal mass. The shape of the growth trajectory of the large *D*. *gazella* thus differs markedly from smaller species that grew for longer, but at a slower rate (Figure 1). From the perspective of life history theory, such negative relationships between age and size at maturity may be shaped by the presence of developmental thresholds (Day & Rowe, 2002; Nilsson‐Örtman & Rowe, 2021; Wilbur & Collins, 1973). Physiological thresholds have indeed been shown to play key roles in arthropod development. For instance, one of these thresholds, critical weight (also called ‘critical size’), refers to the size at which larvae initiate an endocrine cascade that ultimately induces molting and metamorphosis (Mirth & Riddiford, 2007). The exact size threshold value, as well as the amount of left‐over growth able to occur after a given threshold has passed, are able to shape the relationships between age and size at maturity. Moreover, immature stages may encounter multiple such thresholds during ontogeny. In addition, components of developmental thresholds may themselves be dependent upon environmental conditions, employed in a sex‐biased manner, and diverge among species (Ghosh et al., 2013; Rohner et al., 2017). Variation in critical weight (or other) thresholds and associated growth parameters thus likely contributes to defining the relationship between age and size at maturity reported here, yet their precise contributions remain to be determined.

An unexpectedly large contributor to variation in age at maturation was variation in the duration individuals spent in the larval stage *following* the cessation of growth. For instance, while the large *D*. *gazella* only spent 6.2 days between larval peak weight and pupation, the much smaller *L*. *militaris* took 16.7 days on average (Table S1). Evolved differences between species are further reflected in the proportion of time during which larvae grow in the third instar. While *O*. *taurus* grew during 60% of the entire time spent in L3, *L*. *militaris* only grew during 36% of the same stage. This suggests major differences in growth schedules (and consequently the age at maturation) that appear unrelated to the duration of growth itself. In other species, such as butterflies and flies, the period between growth cessation and pupa(ria)tion is referred to as the “wandering stage” during which larvae purge their gut, prepare for metamorphosis, and locate a suitable location for pupation (Dominick & Truman, 1984; Rohner et al., 2017; Sokolowski et al., 1984). As dung beetles do not relocate but pupate inside their brood ball, the function of this stark difference in growth schedules after growth cessation remains unclear. Furthermore, the time spent in the larval stage after growth cessation is unrelated to fluctuating asymmetry in concurrently developing structures, as might have been expected if accelerated development of imaginal tissue leads to developmental instability. Because *L*. *militaris* lacks exaggerated secondary sexual traits, this also seems unlikely to be driven by the development of especially costly adult structures (e.g., the particularly large testes in *Drosophila pachea* [Pitnick, 1993]). The functional relevance and evolutionary implications of this variation therefore await further scrutiny.

In addition to interspecific variation, nutritional quality caused plastic changes in adult size and the shape of the larval growth curves within species. Larvae reared on low‐quality food generally developed slower and extended their development. Yet, while the direction of nutritional effects on age and mass at maturity were similar in all four species, the extent of this plastic response differed strongly among them. This is especially evident when comparing *O*. *binodis*, which exhibited the most pronounced plastic response to nutritional manipulation, to *L*. *militaris*, the least responsive species (Figures 1 and 3). Nutritional plasticity in the *shape* of growth trajectories of *L*. *militaris* also highlight that growth curves are not necessarily related to variation in adult size. Although nutrition affected the shape of the growth trajectory and also total development time (Figure 1), low‐quality nutrition did not reduce adult body size. This finding indicates that the apparent robustness of final body size across nutritional environments is mediated by plastic adjustments of individual growth schedules.

The observations outlined above suggest large variation in the developmental means by which age and size at maturation are determined. This is further reflected in the finding that intrinsic growth rates only correlate with integral growth rates (i.e., crude ratios of adult size and total development time) in two of the four dung beetle species we investigated. Similarly, complex responses in growth curves have been found in other insects (Ghosh et al., 2013; Rohner et al., 2017) and vertebrates (Caccamise & Alexandro, 1976; Ricklefs, 1968). Nevertheless, this complex relationship between growth and age and size at maturity is rarely appreciated in classic life‐history models (van der Have & de Jong, 1996) and comparative evolutionary and ecological studies (Blanckenhorn et al., 2018; Rohner et al., 2016). While this is grounded in a trade‐off between mechanistic detail and predictive simplicity (Davidowitz, 2016; Stearns, 2011), insect growth appears to have more degrees of freedom to vary across species and environments than is sometimes assumed in classic life‐history models (e.g., Abrams et al., 1996; Kozlowski, 1992; Stearns & Koella, 1986).

The precise ultimate selective agents causing the inter‐ and intraspecific variation in life history and growth variation remain unknown. Although the four species possess a similar reproductive biology, feeding ecology, and largely overlapping distribution ranges, there is little comparative data that would allow linking species differences to adaptive or nonadaptive evolutionary processes. Species differences in climatic preferences, diurnal activity patterns, mating systems or associations with other organisms (such as gut bacteria [Parker et al., 2019; Schwab et al., 2016] and nematodes [Ledón‐Rettig et al., 2018]) may account for the evolved differences in age and size at maturation and their relation to growth. Nevertheless, given the large variation in larval growth dynamics documented here, dung beetles appear as an ideal system to investigate the ultimate evolutionary factors driving disparate growth patterns.

### 4.2 How costly is rapid juvenile growth?

Life‐history models predict strong directional selection on growth rate (Roff, 2002); however, growth rate is often found to be lower than physiologically possible. Fast growth rates are thus often argued to be costly, yet identifying these costs is nontrivial (Dmitriew, 2011; Nylin & Gotthard, 1998). We here tested the hypothesis that increasing growth rate comes at the cost of developmental stability, as might be expected due to oxidative stress or pleiotropic effects (Metcalfe & Alonso‐Alvarez, 2010; Nussey et al., 2009; Schäfer et al., 2013; Smith et al., 2016), such that faster growth is expected to increase fluctuating asymmetry (a relationship that has been found in other systems, e.g., Morris et al., 2012; Robinson & Wardrop, 2002). Because the tissue that gives rise to the adult fore tibia is developing during the time when larvae grow most (L3), we expected this functional trait to be affected by such a trade‐off. However, neither tibia size nor tibia shape showed any correlation with instantaneous growth rates. Although this finding does not exclude the possibility that rapid growth affects developmental stability elsewhere, it shows that normative development is possible despite considerable variation in growth rate.

### 4.3 Hidden nutritional plasticity of digestive systems

Plastic and evolved responses of gut morphology have previously been shown to reflect ecological adaptation (Ledón‐Rettig et al., 2008; Wagner et al., 2009). The finding that dung beetle larvae develop larger midguts and spend more time digesting a meal when exposed to a nutritionally challenging diet suggests that larvae invest into a more costly digestive system to enhance nutrient extraction when conditions are poor. Dung beetles may show a particularly strong plastic response relative to more mobile and opportunistic polyphagous insects because—once assessed—food quality is predictable for their entire larval development. Nevertheless, we suspect that developmental plasticity in larval digestive physiology and morphology may often be cryptic and possibly overlooked in assessments of nutritional plasticity, especially in species that are mobile and less specialized in their feeding ecology. Similar patterns have been found in mammals and fish (Kotrschal et al., 2014; Sassi et al., 2007), indicating that plastic and evolutionary diversification of digestive systems may represent a common route of adaptation to nutritional challenges.

We focused on midgut morphology because, in contrast to the fore and hindgut, this section is not lined by cuticle. The lack of cuticular lining allows the midgut to expand during intermolt periods, either through cell proliferation, cell growth, changes in folding, or combinations thereof. In addition, the midgut plays a major role in the production and secretion of digestive enzymes and nutrient absorption (Snodgrass 1994). Both dung beetle species assessed plastically increased gut volume, although *O*. *taurus* enlarged its midgut by increasing its width, while *D*. *gazella* increased both midgut width and length. This hints at divergent developmental mechanisms underpinning nutritional plasticity in species with similar ecological requirements. Furthermore, while both species increase gut volume to a similar extent, *O*. *taurus* had a stronger retention time response to nutritional stress. Given that *O*. *taurus* shows less nutritional plasticity in adult size compared to the larger *D*. *gazella* (see Figure 1), this may indicate that plasticity in gut retention time, rather than gut size, mediates body size plasticity.

These morphological and physiological responses are all the more intriguing because dung beetles are known to depend, to some extent, on the presence of symbiotic gut microbes (Hanski & Cambefort, 1991; Parker et al., 2019; Schwab et al., 2016), and recent studies even hint at species and population differences in the degree of this dependency (Parker et al., 2021). Differences in the means by which digestive systems adjust to nutritional conditions may thus relate to host–endosymbiont interactions. At the same time, dung beetle larvae also continually restructure and physically modify their brood ball environment in a way that benefits their own growth and subsequent adult fitness. Specifically, larvae distribute their own fecal matter within the brood ball and then re‐eat the resulting mixture. These modifications enhance brood ball microbiota's capacity to digest a wide range of carbon sources, raising the possibility that larvae convert brood balls into a type of external rumen, able to predigest brood ball material prior to actual ingestion by larvae. Previous research suggests that the relatively small *O*. *taurus* is considerably more dependent on such brood ball modifying behavior than the much larger *D*. *gazella* (Schwab et al., 2017). Intraspecific variation in growth, and especially the effect of nutrient manipulation, may therefore relate to a species’ reliance on microbiome and niche construction, opening promising avenues for future comparative work on the relationship between digestive plasticity, organism–environment interactions, and life‐history plasticity.

## 5 CONCLUSIONS

Our detailed assessment of larval growth suggests growth schedules to be strongly context‐dependent and evolutionary labile in a nonintuitive manner. This is particularly evident as interspecific differences in development times were largely caused by differences in the time individuals spent in the larval stage after growth cessation. That is, a considerable source of variation in age at adulthood stems from processes unrelated to growth itself. We also found that nutritional adjustments to growth trajectories can mediate robustness of adult size to nutritional conditions. In addition, nutritional plasticity and species differences in gut morphology and physiology may represent an overlooked mechanism contributing to life‐history plasticity in insects. Rarely considered in life‐history and macroecological comparative studies, our data highlight the usefulness and need for detailed assessments of larval growth when studying the mechanisms underlying life‐history evolution.

## CONFLICT OF INTEREST

The authors have no conflicts to declare.

## AUTHOR CONTRIBUTIONS

**Patrick T. Rohner:** Conceptualization (equal); Data curation (equal); Formal analysis (equal); Funding acquisition (equal); Investigation (equal); Methodology (equal); Visualization (equal); Writing‐original draft (equal); Writing‐review & editing (equal). **Armin P. Moczek:** Conceptualization (equal); Funding acquisition (equal); Methodology (equal); Resources (equal); Writing‐review & editing (equal).

## Supporting information

Supplementary Material

## Data Availability

All data underlying this study are available on Dryad (https://doi.org/10.5061/dryad.j9kd51cdc).
