# Supplementary material for: Evolutionary and plastic variation in larval growth and digestion reveal the complex underpinnings of size and age at maturation in dung beetles
Source: Ecol Evol. 2021 Oct 14;11(21):15098–110. doi: 10.1002/ece3.8192 (PMC8571579; doi:10.1002/ece3.8192)
Supplement: Supplementary file 1 — Supplementary Material [file ECE3-11-15098-s001.docx]

**Supplementary material**


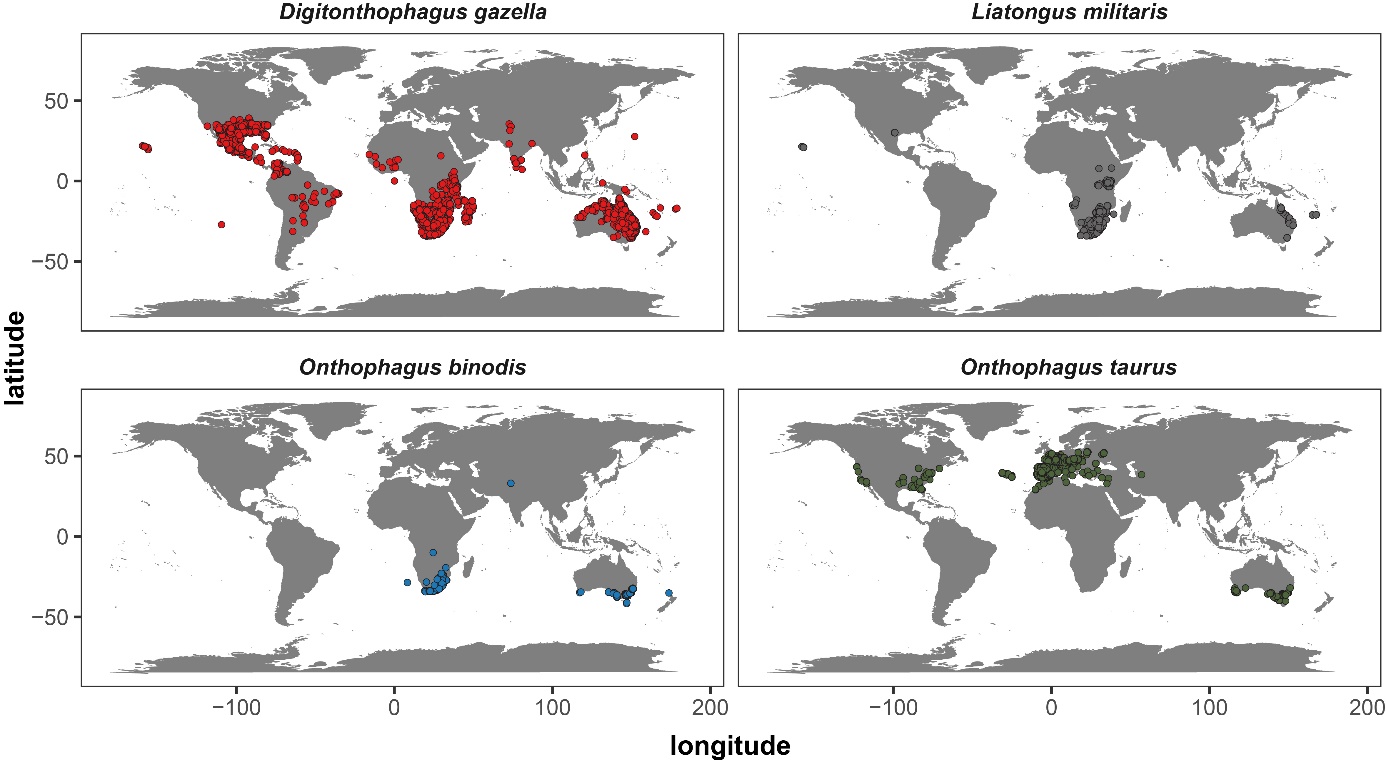


Figure S1: Global distribution of the four investigated species. *D. gazella*, *O. binodis*, and *L. militaris* are native to sub-Saharan Africa while *O. taurus* is native to Central Europe. The exotic (i.e., introduced) ranges of all species broadly overlap, especially in south-eastern Australia where all four species are present. Data from GBIF.org (doi: 10.15468/dl.ep2rep;18 August 2021).


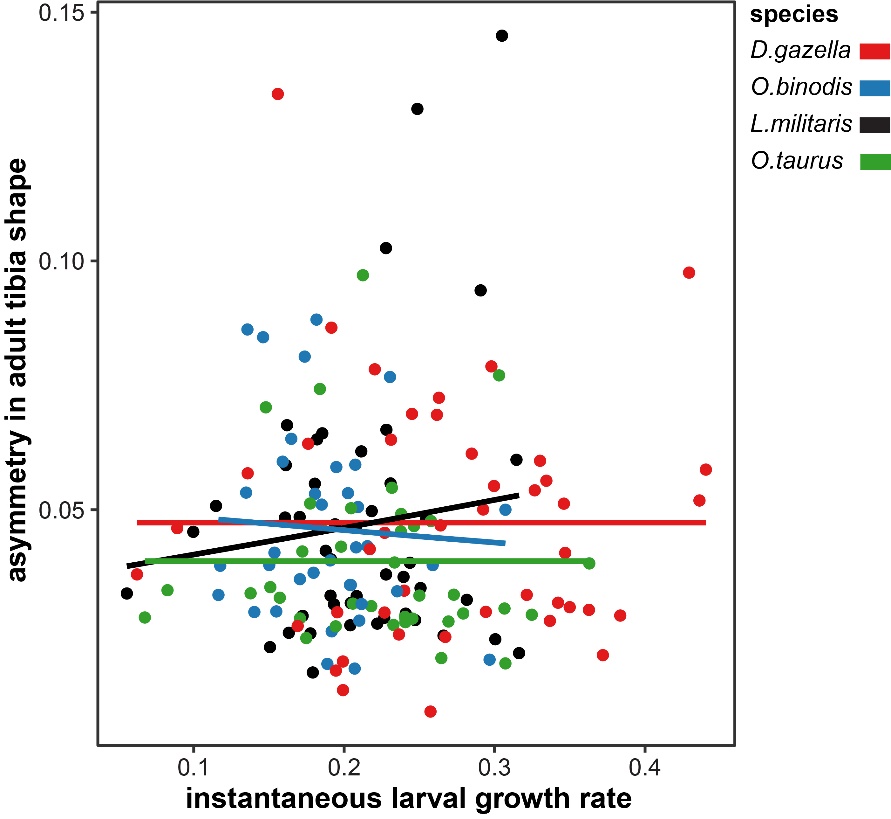


Figure S2: Asymmetry in adult tibia shape does not correlate with instantaneous larval growth rates. Trend lines are derived from generalized additive models.

Table S1: Species- and treatment-specific average age (in days) and body mass (in mg; ± 95% confidence limits) by developmental stage.

|  |  | high-quality nutrition  (dung from grass-fed cows) | | low-quality nutrition  (dung from hay-fed cows) | |
| --- | --- | --- | --- | --- | --- |
| species | **stage** | **age** | **mass** | **age** | **mass** |
| *D. gazella* | L1 | — | 13.6 [11.8,15.5] | — | 13.6 [12.1,15.0] |
|  | L2 | 2.1 [1.9,2.4] | 49.7 [43.9,55.6] | 2.3 [2,2.5] | 48.9 [44.5,53.4] |
|  | L3 | 4.7 [4.4,5.0] | 177.6 [162.9,192.3] | 5.1 [4.9,5.3] | 163.5 [149.2,177.8] |
|  | peak weight | 11.2 [10.5,12] | 450.5 [428.8,472.2] | 12.5 [11.9,13.1] | 419.1 [400.1,438.1] |
|  | pupation | 17.2 [16.6,17.9] | 266.8 [255.5,278] | 18.6 [18.1,19.1] | 213.3 [204.1,222.5] |
|  | adult | 23.7 [23.1,24.3] | 142.8 [130.2,155.3] | 25.5 [25.0,26.0] | 106.4 [96.7,116.2] |
| *L. militaris* | L1 | — | 9.2 [8.3,10.1] | — | 9.7 [8.6,10.9] |
|  | L2 | 4.2 [3.9,4.4] | 38.6 [34.1,43.2] | 4.8 [4.4,5.3] | 39.8 [36.6,43.0] |
|  | L3 | 8.9 [8.6,9.1] | 126.7 [118.3,135] | 9.8 [9.4,10.1] | 122.3 [114,130.6] |
|  | peak weight | 18.0 [17.0,19.0] | 261.0 [252.7,269.3] | 20.4 [18.5,22.2] | 259.2 [250.6,267.9] |
|  | pupation | 33.8 [32.8,34.8] | 131.5 [126.7,136.3] | 37.8 [36.1,39.5] | 138.3 [135,141.7] |
|  | adult | 44.2 [43.2,45.3] | 89.2 [86.3,92.1] | 47.4 [46.3,48.5] | 93.5 [91.4,95.6] |
| *O. binodis* | L1 | — | 19.2 [16.8,21.6] | — | 18.8 [16.6,21.1] |
|  | L2 | 2.5 [2.2,2.7] | 49.8 [45.4,54.2] | 3.3 [2.9,3.6] | 38.9 [36.1,41.7] |
|  | L3 | 6.3 [5.9,6.7] | 143 [134.7,151.3] | 8.7 [7.9,9.5] | 113.7 [105.9,121.6] |
|  | peak weight | 15.7 [14.7,16.6] | 314.8 [304.4,325.2] | 20.8 [19.0,22.5] | 263.5 [243.5,283.5] |
|  | pupation | 25.1 [24.1,26.1] | 182.8 [174.2,191.3] | 32.0 [30.2,33.8] | 146.7 [133.6,159.9] |
|  | adult | 33.6 [32.8,34.5] | 114.5 [107.6,121.4] | 41.1 [38.9,43.3] | 91.8 [79.8,103.9] |
| *O. taurus* | L1 | — | 9.3 [7.8,10.8] | — | 10.3 [9.3,11.3] |
|  | L2 | 2.3 [2.0,2.7] | 24.2 [19.1,29.4] | 2.7 [2.3,3.1] | 20.3 [18.9,21.6] |
|  | L3 | 5.6 [5.0,6.2] | 61.2 [51.8,70.5] | 7.1 [6.6,7.5] | 46.6 [43.4,49.7] |
|  | peak weight | 14.9 [13.8,15.9] | 157.7 [142.4,173] | 17.8 [16.7,18.9] | 128.3 [121.0,135.6] |
|  | pupation | 21.3 [20.5,22.2] | 109.6 [96.6,122.7] | 24.8 [24.0,25.7] | 84.5 [78.0,91.0] |
|  | adult | 29.4 [28.5,30.3] | 73.1 [62.8,83.4] | 32.5 [31.6,33.4] | 55.5 [49.9,61.1] |

Table S2: ANOVA tables (type II SS) of linear models testing for species differences and the effect of larval nutrition (hay or grass dung) on the relationship between growth parameters and adult size estimated as logarithmized pronotum width.

| **Adult size vs. egg-to-adult development time** | | | | | | |
| --- | --- | --- | --- | --- | --- | --- |
|  | SS | df | F | P | η^2^ | η^2^_ρ_ |
| **total development time** | 5.30E-03 | 1 | 1.76 | 0.187 | <.01 | 0.01 |
| **species** | 1.92E+00 | 3 | 212.32 | <.001 | 0.77 | 0.81 |
| **larval nutrition** | 3.90E-02 | 1 | 12.93 | <.001 | 0.02 | 0.08 |
| **total development time x species** | 2.26E-02 | 3 | 2.5 | 0.062 | 0.01 | 0.05 |
| **species x larval nutrition** | 3.80E-02 | 3 | 4.2 | 0.007 | 0.02 | 0.08 |
| **residuals** | 4.59E-01 | 152 |  |  |  |  |
|  |  |  |  |  |  |  |
| **Adult size vs. duration of larval growth** | | | | | | |
|  | SS | df | F | P | η^2^ | η^2^_ρ_ |
| **duration larval growth period** | 5.50E-03 | 1 | 1.77 | 0.185 | <.01 | 0.01 |
| **species** | 1.64E+00 | 3 | 176.06 | <.001 | 0.71 | 0.76 |
| **larval nutrition** | 7.90E-02 | 1 | 25.42 | <.001 | 0.03 | 0.13 |
| **species x larval nutrition** | 8.96E-02 | 3 | 9.61 | <.001 | 0.04 | 0.15 |
| **residuals** | 5.07E-01 | 163 |  |  |  |  |
|  |  |  |  |  |  |  |
| **Adult size vs. weight lost after larval peak weight** | | | | | | |
|  | SS | df | F | P | η^2^ | η^2^_ρ_ |
| **log weight lost after larval peak** | 3.98E-02 | 1 | 13.74 | <.001 | 0.03 | 0.08 |
| **species** | 6.58E-01 | 3 | 75.72 | <.001 | 0.47 | 0.58 |
| **larval nutrition** | 1.24E-01 | 1 | 42.81 | <.001 | 0.09 | 0.21 |
| **species x larval nutrition** | 1.03E-01 | 3 | 11.86 | <.001 | 0.07 | 0.18 |
| **residuals** | 4.72E-01 | 163 |  |  |  |  |
|  |  |  |  |  |  |  |
| **Adult size vs. pupal weight** | | | | | | |
|  | SS | df | F | P | η^2^ | η^2^_ρ_ |
| **log pupal weigth** | 5.11E+00 | 1 | 2360.86 | <.001 | 0.81 | 0.94 |
| **species** | 7.94E-01 | 3 | 122.12 | <.001 | 0.13 | 0.69 |
| **larval nutrition** | 4.00E-04 | 1 | 0.18 | 0.674 | <.01 | 0 |
| **log pupal weight x species** | 1.82E-02 | 3 | 2.81 | 0.041 | <.01 | 0.05 |
| **residuals** | 3.53E-01 | 163 |  |  |  |  |
|  |  |  |  |  |  |  |
| **Adult size vs. instantaneous growth rate** | | | | | | |
|  | SS | df | F | P | η^2^ | η^2^_ρ_ |
| **growth rate** | 1.51E-03 | 1 | 0.49 | 0.487 | 0 | 0 |
| **species** | 2.39E+00 | 3 | 255.81 | <.001 | 0.77 | 0.83 |
| **larval nutrition** | 1.08E-01 | 1 | 34.55 | <.001 | 0.03 | 0.18 |
| **growth rate x species** | 2.17E-02 | 3 | 2.32 | 0.077 | 0.01 | 0.04 |
| **growth rate x larval nutrition** | 9.38E-03 | 1 | 3.01 | 0.085 | <.01 | 0.02 |
| **species x larval nutrition** | 8.99E-02 | 3 | 9.63 | <.001 | 0.03 | 0.16 |
| **residuals** | 4.89E-01 | 157 |  |  |  |  |

Table S3: ANOVA tables (type II SS) of linear models testing for a relationship between fluctuating asymmetry and growth rate and the time spent in the third instar after the cessation of growth. Interactions with larval nutrition (hay or grass dung) and species identity were non-significant and removed.

| Fluctuating asymmetry in tibia centroid size | | | | |  | Fluctuating asymmetry in tibia centroid size | | | | |
| --- | --- | --- | --- | --- | --- | --- | --- | --- | --- | --- |
|  | **SS** | **df** | **F** | **P** |  |  | **SS** | **df** | **F** | **P** |
| instantaneous rowth rate | 2.97E-05 | 1 | 0.63 | 0.428 |  | **time spent in L3 after peak weight** | 1.66E-05 | 1 | 0.36 | 0.551 |
| species | 1.94E-04 | 3 | 1.37 | 0.253 |  | **species** | 1.40E-04 | 3 | 1.00 | 0.395 |
| larval nutrition | 1.95E-05 | 1 | 0.41 | 0.521 |  | **larval nutrition** | 1.49E-05 | 1 | 0.32 | 0.572 |
| residuals | 7.49E-03 | 159 |  |  |  | **residuals** | 7.50E-03 | 161 |  |  |
| Fluctuating asymmetry in tibia shape | | | | |  | **Fluctuating asymmetry in tibia shape** | | | | |
|  | **SS** | **df** | **F** | **P** |  |  | **SS** | **df** | **F** | **P** |
| instantaneous growth rate | 3.20E-05 | 1 | 0.06 | 0.803 |  | **time spent in L3 after peak weight** | 6.15E-04 | 1 | 1.20 | 0.275 |
| species | 1.67E-03 | 3 | 1.10 | 0.353 |  | **species** | 2.12E-03 | 3 | 1.38 | 0.250 |
| larval nutrition | 3.82E-04 | 1 | 0.75 | 0.388 |  | **larval nutrition** | 3.60E-04 | 1 | 0.70 | 0.403 |
| residuals | 8.10E-02 | 159 |  |  |  | **residuals** | 8.24E-02 | 161 |  |  |
